# Timing of inotropic support is associated with mortality in patients with acute decompensated heart failure-associated cardiogenic shock

**DOI:** 10.1186/s40635-025-00806-z

**Published:** 2025-10-31

**Authors:** John C. Greenwood, Charith Ratnayake, Moizza Shabbir, Samantha Opitz, David H. Jang, Wook-Jin Choi, Nova L. Panebianco, Frances S. Shofer, John G. T. Augoustides, Jan Bakker, Joyce W. Wald, Benjamin S. Abella

**Affiliations:** 1 Department of Emergency Medicine, Center for Resuscitation Science, Perelman School of Medicine at the University of Pennsylvania, Philadelphia, PA USA; 2 Department of Anesthesiology and Critical Care, Perelman School of Medicine at the University of Pennsylvania, Philadelphia, PA USA; 3 Department of Emergency Medicine, Ulsan University Hospital, Ulsan, Korea; 4 Department of Epidemiology & Biostatistics, Department of Emergency Medicine Hospital of the University of Pennsylvania, Philadelphia, PA USA; 5 Department of Intensive Care Adults, Erasmus MC University Medical Center, Rotterdam, The Netherlands; 6 Division of Cardiovascular Medicine, Department of Medicine, Perelman School of Medicine at the University of Pennsylvania, Philadelphia, PA USA; 7 Department of Emergency Medicine, Icahn School of Medicine at Mount Sinai, New York, NY USA

**Keywords:** Acute decompensated heart failure, Cardiogenic shock, Hospital mortality, Resuscitation

## Abstract

**Background:**

Inotropic support is often used to improve hemodynamics and organ perfusion in patients with advanced heart failure-related cardiogenic shock (ADHF-CS). We aimed to evaluate the effect of inotrope timing on patient mortality in patients meeting Society for Cardiovascular Angiography and Interventions (SCAI) stage-C criteria within 24 h of hospital presentation.

**Methods:**

We analyzed a local cardiogenic shock database of patients admitted to our cardiovascular intensive care units at the University of Pennsylvania from five emergency departments between 2021 and 2023. Adult patients with left ventricular ejection fraction ≤40% were eligible for inclusion. Patients with hypoperfusion, who met at least one physical, biochemical, and hemodynamic criterion for SCAI-C shock were included. The primary outcome was 28-day mortality. We also compared SCAI criteria and diagnostic examination timing between early and delayed inotropic support groups.

**Results:**

A total of 138 out of 623 patients (22%) with cardiogenic shock met inclusion criteria for this study. 28-day mortality was higher in patients who received inotropic therapies ≥8 h after cardiogenic shock onset compared to patients who received earlier support (4-h odds ratio of death (OR) 3.19, 95% CI: 1.34–8.03; 8-h OR: 2.4, 95% CI: 1.09–5.26). 28-day mortality was lower in the early inotrope group (<8 h from shock onset) compared to the delayed (≥8 h) group (15/87; 17% vs. 17/51; 32%, *p* = 0.031). Patients with early inotropic support more often presented with a cool peripheral exam (34% vs. 16%, *p* = 0.022) and an initial lactate > 2 mmol/dL (71% vs. 49%, *p* = 0.009). Delayed inotropic support was associated with hypotension at presentation (84% vs. 57%, *p* = 0.001), longer time to echocardiography (19 [11–36] vs. 15 [3–24] h, *p* = 0.053) and time to pulmonary artery catheterization (25 [16–45] vs. 16 [2–46] h, *p* = 0.042).

**Conclusion:**

Our findings suggest that inotropic support initiated within 8 h of acute presentation is associated with decreased 28-day mortality for patients with ADHF-related cardiogenic shock. Peripheral perfusion and cardiac output measurement were less frequently quantified within the first 24 h for patients with delayed inotropic support. Using shock classification tools, such as the SCAI shock criteria, may help identify patients with CS, especially in its early stages.

**Supplementary Information:**

The online version contains supplementary material available at 10.1186/s40635-025-00806-z.

## Introduction

Acute decompensated heart failure (ADHF) is the leading cause of cardiogenic shock (CS) in the United States and European countries and is associated with a 30–50% mortality [1, 2]. Historically, acute myocardial infarction (AMI) was the most common cause of CS, and much of the evidence for the treatment of modern CS was derived from the AMI patient population. But over the past 15 years, the incidence of ADHF-CS has increased and is now responsible for nearly 40% of all CS admissions [3]. The epidemiologic shift is significant, as very little evidence exists to guide resuscitation strategies for ADHF-CS [4, 5]. In the United States, cardiogenic shock mortality has remained unchanged over the past 15 years. Moreover, the decline in heart failure-related mortality seen between 1999 and 2012 has been completely reversed between 2012 and 2021—resulting in higher heart failure mortality in 2024 than in 1999 [6].

Therapeutic interventions for time-sensitive critical illnesses such as door-to-balloon time in ST elevation myocardial infarction, antibiotics in sepsis, and thrombolytics in ischemic stroke have been recognized as a critical component of high-quality care and improved outcomes [7–9]. Organ reperfusion by increasing cardiac output is often the first step in the treatment of CS, but data on inotrope timing and clinical factors that may contribute to delays in starting inotropic support are poorly described in the literature. Recent trials have compared the effect of different inotropes on patient outcomes, but the effect of time between shock onset and inotropic therapy has not been explored [10, 11].

The aim of this study was to evaluate the relationship between the timing of inotropic therapy relative to the onset of cardiogenic shock and hospital mortality, as well as examine clinical factors that are associated with delays in inotropic therapy for patients presenting with ADHF-CS.

## Materials and methods

### Study design and setting

We performed a retrospective, observational cohort study using a local cardiogenic shock database of patients in the University of Pennsylvania Health System who presented to one of five Emergency Departments (ED) and were admitted to a cardiovascular intensive care unit or step-down unit with a diagnosis of cardiogenic shock between 1/1/2021 and 12/31/2023. The study was approved by the University of Pennsylvania Institutional Review Board (protocol #855463) on 3/1/2024 and included a waiver of informed consent. We identified eligible subjects from a local cardiogenic shock database search using the *International Classification of Diseases, Ninth Revision (ICD-9; 785.51)* or *ICD-10 (R57.0)* code for cardiogenic shock. All management decisions, including the use of pulmonary artery catheterization, were made at the discretion of the treating clinicians based on clinical judgment for hemodynamic monitoring when deemed necessary. We then manually reviewed records for patients who met Society for Cardiovascular Angiography & Interventions (SCAI) class C shock within 24 h of ED presentation to abstract missing data when available (Supplemental Table 1).

### Selection of participants and definitions

Adult patients (>8 years old) with left ventricular ejection fraction ≤40% by echocardiogram (new or previously documented) were eligible for inclusion. Patients with hypoperfusion, identified by abnormalities in physical examination, biochemical, and hemodynamic criteria along with the need for pharmacologic or mechanical support, were classified as SCAI class C shock [12]. Patients who were transferred from an outside hospital inpatient service, determined to have a non-cardiogenic cause of shock, out-of-hospital cardiac arrest, ST-elevated myocardial infarction, heart failure with a preserved left ventricular ejection fraction, receiving home inotropic therapy, had a left ventricular assist device, an acute valvular etiology, shock due to a post-operative complication, or heart transplant were excluded from the study. Inotropic support was defined as the initiation of dobutamine, milrinone, or epinephrine infusion. These agents were grouped based on their predominant intended clinical use to augment cardiac output at our institution, rather than relying solely on pharmacologic mechanism-based classification. Time to initial administration of inotropic support was calculated as the duration of time between meeting criteria for SCAI class C criteria and the initiation of one of these agents in the medication administration record. Time to cardiac output measurement was defined by the first method to measure cardiac output (echocardiography or pulmonary artery catheterization). To quantify the intensity of vasoactive support within the first 24 h of shock presentation, the vasoactive-inotropic score (VIS) was calculated as follows: VIS = dobutamine + dopamine + 10 × phenylephrine + 10 × milrinone + 100 × epinephrine + 100 × norepinephrine + 10,000 × vasopressin, where doses for all agents are in μg/kg/min with the exception of vasopressin, which is in units/kg/min [13].

### Data collection and processing

**Fig. 1:**
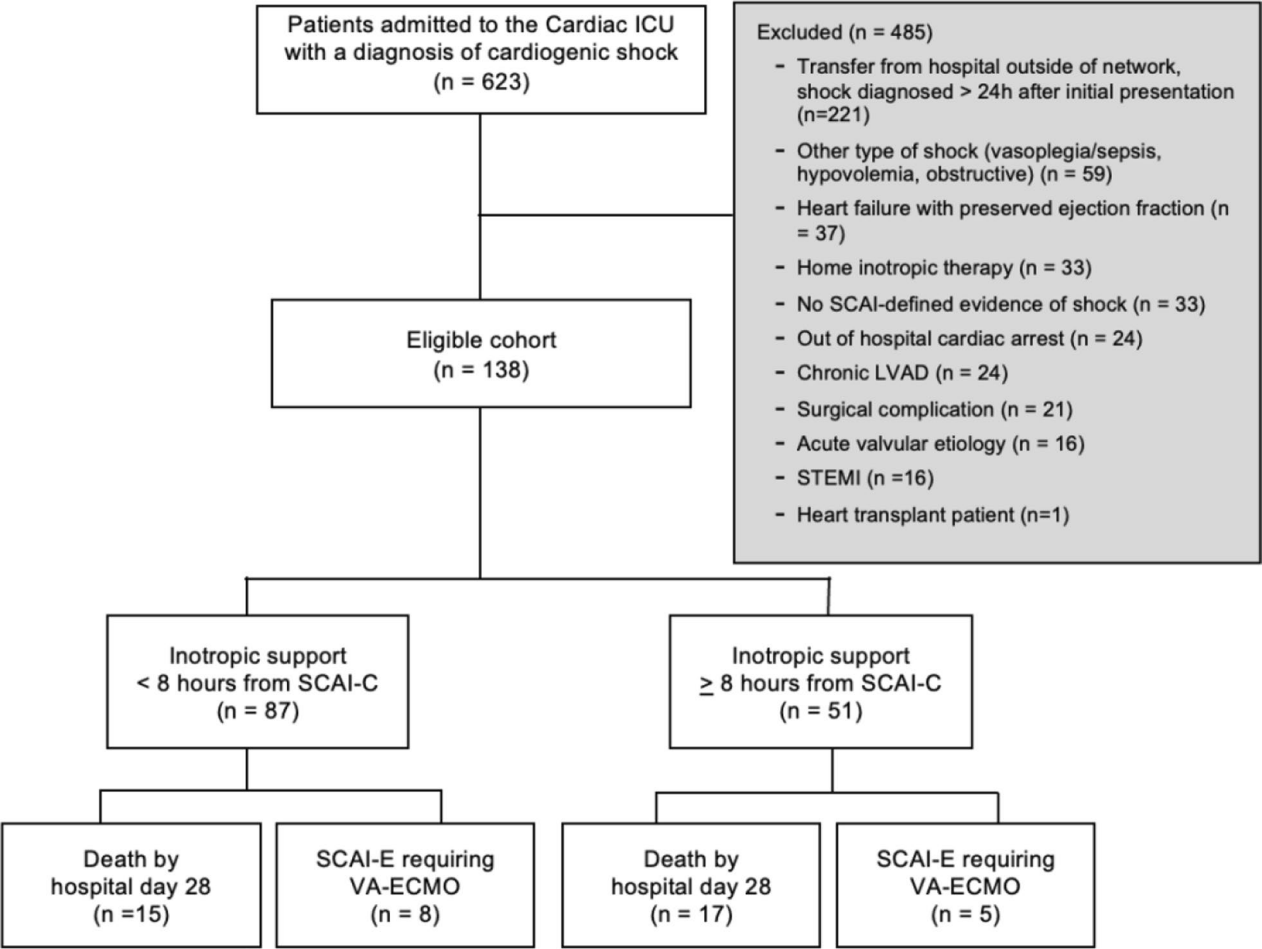
STROBE flowchart for patient inclusion and exclusion

Records were individually reviewed by one of four investigators (JCG, CR, MS, and SO) and study-specific data were abstracted into an internet-accessible, encrypted database (REDCap, Vanderbilt University, Nashville, TN) hosted at the University of Pennsylvania [14, 15]. We collected details in regard to SCAI shock class, timing of cardiogenic shock interventions, serial hemodynamic and perfusion variables, vasoactive administration, echocardiographic data, right heart catheterization data, and clinical outcomes. All patients included in the final analysis had complete baseline data regarding physical examination findings, biochemical markers, and hemodynamic variables necessary for SCAI Stage C classification. Patients with missing critical data at baseline were excluded during the initial screening process, as detailed in the “Selection of participants and definitions” section and shown in Fig. 1. Therefore, no imputation or additional handling of missing data was required during the analysis. Ten percent of records were co-reviewed by the PI (JCG) to ensure inter-rater reliability was >90% using the Bland–Altman method. Statistical analysis was performed with GraphPad Prism version 10.2.3 (Boston, MA).

### Outcome measures

The primary outcome was 28-day mortality. Secondary outcomes included need for venoarterial extracorporeal membrane oxygenation (VA-ECMO), a composite outcome which included both mortality and VA-ECMO, time from SCAI Stage C onset to echocardiography, and time to pulmonary artery catheterization. Time cutoffs for early and late inotrope support were based on an odds ratio (OR) analysis. We compared patient demographics, hemodynamics, SCAI criteria, echocardiography data, PAC data, and perfusion data between groups.

### Data analysis

We first performed an odds ratio analysis using 4-h time blocks to examine the effect of time-to-inotropic therapy on 28-day mortality in patients presenting with SCAI-C stage ADHF-CS. We used the results of this analysis to define early vs. late treatment groups. We chose the 8-h cutoff pragmatically as the longest interval associated with a statistically significant survival difference, while maintaining clinical relevance to typical Emergency Department length of stay.

Data were assessed for normality using the D’Agostino and Pearson omnibus normality test. Normally distributed variables were expressed as means with ±SD. Variables that were not normally distributed are reported as median with interquartile range [25th–75th percentiles]. Continuous variables were compared using Student *t* test or Mann–Whitney *U* test. Categorical variables were compared using the chi-square or Fisher’s exact test. For categorical variables with small counts (*n* < 5), such as race, a global Fisher’s Exact Test was performed to compare overall group distributions. All comparisons were unpaired, and *p* values were two-tailed. All *p* values are listed at the 0.001 level, and a value of <0.050 was considered statistically significant.

## Results

### Patient demographics

A total of 623 subjects were admitted to our hospitals’ cardiac intensive care units with the diagnosis of cardiogenic shock. After an initial review, 485 subjects met exclusion criteria, leaving 138 subjects eligible for the study (Fig. 1).

Inotropic therapy was administered in <8 h of meeting SCAI-C shock criteria in 87 subjects (early group) and ≥8 h in 51 subjects (delayed group). Subject demographics are shown in Table 1.

**Table 1:** Patient demographics and characteristics

Characteristics	Early inotrope < 8 h (*n* = 87)	Late inotrope ≥ 8 h (*n* = 51)	*p* value
Age (years)	61 ± 15	59 ± 16	0.394
Gender, male (%)	61 (70)	26 (51)	0.189
Race *n*, (%)			0.126
White	26 (30)	22 (43)	
Black	54 (62)	27 (53)	
Other	7 (8)	1 (2)	
Comorbidities *n*, (%)			
Non-ischemic cardiomyopathy	65 (75)	27 (53)	0.052
Ischemic cardiomyopathy	22(25)	21 (41)	0.986
Diabetes	28 (32)	12 (24)	0.279
Chronic kidney disease	29 (33)	13 (25)	0.334
LVEF %	20 [14-28]	20 [15–30]	0.340
APACHE II score	18.5 ± 9.4	20.5 ± 8.1	0.207
Hospital LOS (days)	19 [14–30]	20 [14–30]	0.637

Patient demographics were generally similar between early and delayed treatment groups, including race, gender, comorbidities, left ventricular ejection fraction, and APACHE II Score.

### SCAI cardiogenic shock criteria at presentation

Qualifying SCAI-C criteria are shown in Table 2.

**Table 2:** SCAI shock Stage C criteria for patients presenting with cardiogenic shock

SCAI shock C criteria	Early inotrope < 8 h (*n* = 87)	Late inotrope > 8 h (*n* = 51)	*p* value
Physical exam criteria, *n* (%)			
Peripheral exam—cool extremities	29 (33)	8 (16)	0.022
Was capillary refill time measured? Yes	19 (22)	14 (27)	0.456
Altered mental status	13 (15)	6 (12)	0.601
Invasive or non-invasive mechanical ventilation	15 (17)	6 (12)	0.387
Urine output < 30 mL/h	5 (6)	5 (10)	0.375
Volume overload	70 (80)	44 (86)	0.384
Biochemical markers, *n* (%)			
Lactate > 2 mmol/dL	62 (71)	25 (49)	0.009
>50% decrease in GFR	40 (46)	20 (39)	0.439
Abnormal liver function tests	25 (29)	18 (35)	0.422
Elevated B-natriuretic peptide	59 (68)	35 (69)	0.921
Hemodynamic criteria met first, *n* (%)			
SBP < 90 or MAP < 60 mmHg	50 (57)	43 (84)	0.001
Cardiac index < 2.2 L/min/m^2^	34 (39)	12 (24)	0.061

The most common physical exam findings in patients with cardiogenic shock were volume overload (82.6%) followed by cool extremities on peripheral exam (26.8%). Capillary refill time was only measured in 19.1% of patients. An elevated B-natriuretic peptide level (68.1%) and lactate >2 mmol/dL (63.0%) were the most common biochemical criteria met for SCAI-C shock criteria. Hypotension, defined by a systolic blood pressure < 90 mmHg or mean arterial pressure < 65 mmHg (67%), was the most common hemodynamic criteria met for class C shock. Early inotropic support for patients with ADHF-CS was associated with an abnormal peripheral perfusion exam (35% vs. 16%, *p* = 0.022) and abnormal lactate (71% vs. 49%, *p* = 0.009) on presentation. Hypotension was more common in the delayed inotropic therapy group (84% vs. 57%, *p* = 0.001).

### Main results

**Fig. 2:**
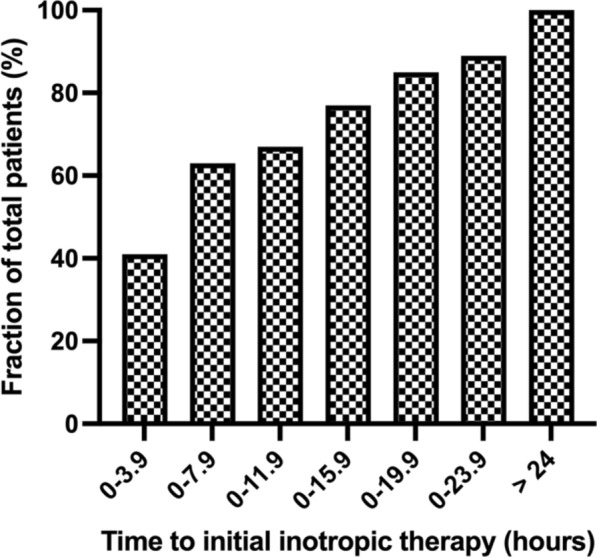
Cumulative representation of the percent of patients to receive inotropic therapy by the indicated upper limit of time

Overall, 28-day mortality was 17% (15/87 patients) in the early inotrope group, and 33% (17/51 patients) in the delayed group (*p* = 0.031). The median duration of time between shock onset and inotropic therapy administration was 5.0 [2.0–14.0] h. Milrinone was the most commonly used inotrope (53.6%), followed by dobutamine (27.5%) and epinephrine (24.6%). Fifteen patients received more than one inotropic infusion during the first 24 h of resuscitation. Norepinephrine was the most commonly used vasopressor (37.7% of patients). In the early inotrope group, 24/87 (28%) of patients required both norepinephrine and inotropic therapy; 0 received norepinephrine alone. In the late inotrope group, 26/51 (51%) of patients required norepinephrine and inotropic therapy; 3 patients received norepinephrine only. A total of 41% of patients received inotropic therapy within 4 h of meeting SCAI-C cardiogenic shock criteria, and 59% within 8 h (Fig. 2).

Patients with early inotropic therapy received less vasopressor therapy (34% vs. 67%, *p* < 0.001), and the delayed inotrope group more often received vasopressors prior to inotropic therapy (45% vs. 11%, *p* < 0.001). Cumulative vasoactive use was higher in the delayed inotrope group (VIS 7.1 [1.3–21.3] vs. 2.5 [1.3–9.2], *p* = 0.013).

**Fig. 3:**
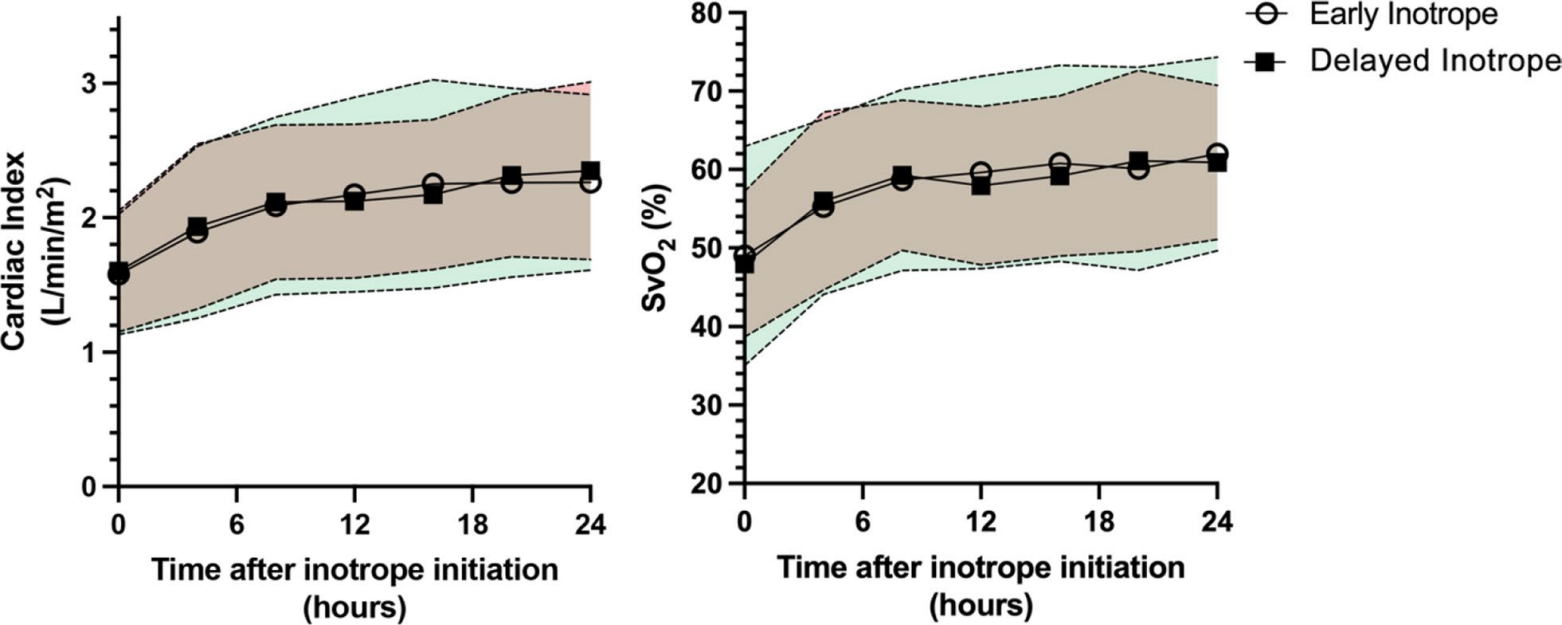
Response to inotropic therapies during the first 24 h of resuscitation (mean, SD). There was no difference in changes to cardiac index or mixed venous oxygen saturation between early and delayed inotropic support groups

Continuous cardiac output and mixed venous oxygen saturation were measured by pulmonary artery catheter in 46/87 early inotrope patients and 31/51 late inotrope patients. Response to inotropic therapy was similar between both groups over the first 24 h of treatment, and there was no difference in cardiac index or mixed venous oxygen saturation over time (Fig. 3).

Time to cardiac output measurement was shorter in patients who received early inotropic support but had a wide variability (6.5 [1.0–20.8] vs. 18.5 [8.5–24.2] h, *p* < 0.001). Similar trends were observed for time to echocardiography and invasive hemodynamic monitoring by PAC (Table 3).

**Table 3:** Clinical investigations, interventions, and outcomes in patients with ADHF-CS

	<8 h (*n* = 87)	≥8 h (*n* = 51)	*p* value
Diagnostic investigations			
Time to cardiac output measurement (h)	6.5 [1.0–20.8]	18.5 [8.5–24.2]	<0.001
Time to transthoracic echocardiography (h)	15 [3–24]	19 [11–36]	0.053
Time to pulmonary artery catheter (h)	16 [2–46]	25 [16–45]	0.042
Vasoactive requirements			
Vasopressor plus inotrope, *n* (%)	30 (34)	34 (67)	<0.001
Vasopressor started prior to inotrope, *n* (%)	10 (11)	23 (45)	<0.001
Vasoactive-inotropic score (VIS, first 24 h)	2.5 [1.3–9.2]	7.1 [1.3–21.3]	0.014
Hemodynamics			
Heart rate (bpm)	105 ± 24	109 ± 37	0.503
Stroke volume (mL) by echocardiography	34 [24–43]	35 [28–53]	0.288
Cardiac index (L/min/m^2^)	1.6 [1.3–2.0]	1.6 [1.3–2.1]	0.585
Central venous pressure (mmHg)	15 [8–15]	15 [8–15]	0.322
Systemic vascular resistance index (dynes/sec/cm^5^/m^2^)	3186 ± 1262	3080 ± 1311	0.658
Perfusion metrics			
Peak lactate (mmol/L)	3.6 [2.1–5.3]	2.6 [1.4–5.8]	0.475
Initial SvO_2_%	53 ± 13	52 ± 12	0.661
Outcomes			
28-day mortality, *n* (%)	15 (17)	17 (33)	0.031
VA-ECMO, *n* (%)	8 (9)	5 (10)	0.906

**Fig. 4:**
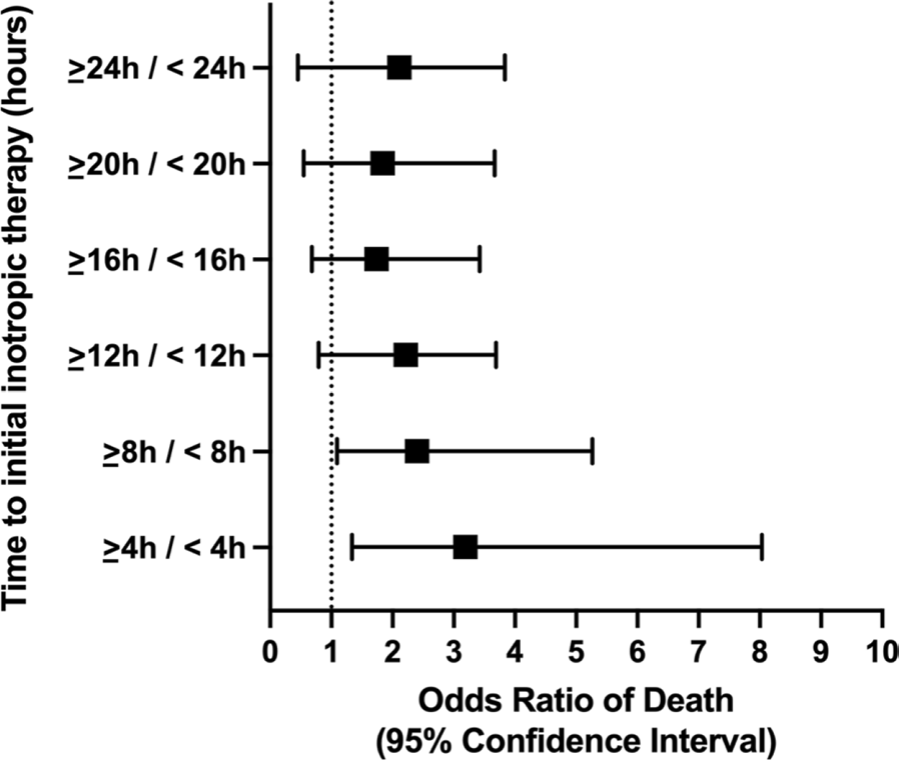
Relationship between different inotropic therapy delays after onset of cardiogenic shock and risk of 28-day mortality expressed as odds ratio of death. Bars represent 95% confidence interval

The odds ratio of death (OR) was significant at 4 h (3.19, 95% CI: 1.34–8.03) and 8 h (OR: 2.4, 95% CI: 1.09–5.26) compared to longer time intervals as shown in Fig. 4.

## Discussion

The data in this study show that the duration of time between onset of ADHF-CS and inotrope administration is an important determinant of patient survival. The risk of poor clinical outcome was highest in patients where the delay in inotrope therapy was greater than 4 h, but also significant at the 8-h cutoff. Patients with delayed inotrope initiation had a longer duration of time between onset of shock and critical diagnostic tests (including echocardiography, pulmonary artery catheterization, and cardiac output measurement) and more commonly presented with hypotension. In contrast, patients who received early inotropic therapy more often presented with an abnormal peripheral exam and abnormal lactate.

This study is an important investigation of the effect of inotropic support timing on clinical outcomes in patients with ADHF-CS. Our analysis revealed that patients receiving early inotrope administration (within 8 h of meeting SCAI-C criteria) had a lower vasopressor requirement, higher peak lactate levels, and were associated with reduced mortality, despite similar severity of illness scores and baseline cardiac function between groups. Augmenting cardiac output to restore organ perfusion is a critical management step in ADHF-CS, yet data on inotrope choice and timing are limited. Current guidelines and expert statements recommend using vasoactives to normalize systemic hemodynamics, but evidence on how quickly these medications should be administered or what target they should be titrated to achieve is scarce [1, 16]. Milrinone was the most common inotrope chosen in our cohort, which likely reflects institutional preference and a hemodynamically tailored decision based on the high systemic vascular resistance measured by echocardiography or pulmonary artery catheterization. A lack of emphasis on timing may contribute to the persistently high mortality rates observed in CS clinical trials. For instance, the DOREMI trial found no difference in mortality between patients assigned to milrinone vs. dobutamine, and reported a 37–43% mortality rate similar to other trials and registries [11, 17]. DOREMI trial patients were similar to our cohort, as 80% of patients met SCAI-C criteria, but randomization to the study drug only occurred after ICU admission, which likely delayed inotrope initiation. The significant odds ratios for our primary outcome suggest that initiating inotropic therapy within 8 h of CS onset (which is also an important ED-centric time frame) may reduce patient mortality.

Time to inotropic therapy was analyzed in 4-h blocks to pragmatically visualize survival trends, and an 8-h cutoff was selected to define early versus late groups, based on observed survival differences. This decision was made considering the sample size limitations and the exploratory nature of the study. Consistently, patients who received early inotropic therapy demonstrated lower cumulative vasoactive requirements, which may reflect earlier hemodynamic stabilization and highlight the potential importance of prompt intervention in ADHF-CS. Importantly, the fact that the early inotrope group demonstrated statistically significant differences in vasopressor requirements, time to cardiac output measurement, and 28-day mortality reinforces the overall robustness of this study.

In our cohort, patients with delayed inotrope administration more commonly received norepinephrine as their first vasoactive treatment, which may have normalized blood pressure but delayed the introduction of inotropic therapies. We suspect this likely occurred because hypotension is more easily identified and treated in the ED setting. Advanced echocardiography, pulmonary artery catheterization, and cardiac output monitoring that can identify low cardiac output are generally not available in Emergency Departments, which likely impact time-based decisions on inotropic initiation for ADHF-CS in the early presentation period. Clinicians should be aware that normalized macrocirculatory variables do not always guarantee adequate perfusion, as microcirculatory dysfunction may persist even with normalized blood pressure in patients with cardiogenic shock [18, 19]. Hypotension in patients with ADHF-CS is a complex hemodynamic variable, as it may represent inadequate cardiac output or systemic vasoplegia (mixed shock), requiring different therapeutic interventions. Moreover, conventional shock biomarkers such as lactate, renal function tests, and mixed venous oxygen may not sufficiently distinguish early versus late shock states.

We utilized the SCAI cardiogenic shock classification to standardize the definition of cardiogenic shock and estimate patient mortality, which were originally developed as a retrospective risk stratification tool and rely on evolving clinical and hemodynamic parameters that may not be fully apparent at initial presentation. Alternative CS prediction tools, such as the SHARC criteria (Simple Hemodynamic Assessment to Risk-Stratify Cardiogenic Shock) developed specifically to identify cardiogenic shock in real time, were designed for clinical environments where timely recognition of CS is critical and where full hemodynamic data may not yet be available [20]. Given the high percentage of patients with isolated hypoperfusion, normal blood pressure, and low cardiac output identified in our cohort, SHARC may fill a critical gap in the early recognition and triage of cardiogenic shock in the ED, enabling more timely and targeted interventions.

Although elevated lactate was the most common biomarker meeting SCAI-C shock criteria, it may not be the most reliable indicator of poor tissue perfusion or mortality risk in ADHF-CS. While often used as a marker of tissue hypoxia, lactate levels can also rise due to factors like high central venous pressure, vasoconstriction, sympathetic activation, low oxygen levels, anemia, or liver and kidney dysfunction. In some cases, lactate may be less sensitive than other indicators of impaired perfusion, such as capillary refill time (CRT) [21]. The higher frequency of cold extremities observed in early inotrope patients may be reflective of a patient group whose physiology had not yet progressed to compensatory vasodilation or a mixed shock state. Subjective assessments of peripheral perfusion may be less preferred compared to qualitative measures of peripheral perfusion. Capillary refill time is a low-cost, easily measured surrogate for microcirculatory blood flow but is not routinely used or studied as a therapeutic trigger/target in CS patients [22, 23]. In our study, CRT was documented in <30% of patient encounters. Future studies that evaluate the effect of inotropic support on peripheral perfusion may provide a non-invasive perfusion target to help clinicians titrate inotropic therapies.

Pulmonary artery catheterization and echocardiography are essential diagnostic tools that help characterize a patient’s hemodynamic phenotype. While ADHF-CS is defined by low cardiac output, other factors such as ventricular preload (central venous pressure or pulmonary capillary wedge pressure), volume status, and systemic vascular resistance vary by patient. An early hemodynamic profile with echocardiography or PAC could help define the patient’s shock state and guide early interventions [24]. Time from shock onset to echocardiography and PAC placement was shorter in the early inotrope group, which highlights their value in providing actionable clinical data to guide CS therapies. It is important to note, however, there was a large variability in how long it took to obtain these data. This likely reflects variations in clinical practice but could also be a result of differences in specialist expertise, procedural comfort, and the duration of time between Emergency Department presentation and hospital admission (ED boarding time). Interestingly, improvement in cardiac output and mixed venous oxygen saturation were similar between early and late groups, despite the difference in mortality. Increased mortality may be the result of prolonged, inadequate organ perfusion leading to multisystem organ failure or hemometabolic phenotype, which has been associated with a higher mortality rate in patients with acute cardiogenic shock [25]. A standardized approach to clinical care in patients with ADHF-CS, which includes early echocardiography and hemodynamic phenotyping, could reduce time to critical reperfusion therapies.

Finally, one-third of ADHF-CS patients were normotensive despite clear evidence of hypoperfusion, a higher incidence than expected. Normotensive cardiogenic shock is a recognized hemodynamic phenotype of CS, estimated to occur in 5–20% of patients with acute CS, and is associated with a significantly higher patient mortality [26, 27]. Hypotension alone has been linked to a moderate increase in mortality, whereas hypoperfusion, whether isolated or combined with hypotension, markedly increases mortality risk [1]. These findings suggest that patients with normotensive hypoperfusion may be paradoxically sicker at the time of presentation, reinforcing the internal validity of our data. One hypothesis is that early on during acute cardiogenic shock, compensatory mechanisms maintain blood pressure despite declining cardiac output, which masks the severity of shock. As the natural history of ADHF-CS progresses, vasoplegia may become more prominent, leading to a decline in diastolic blood pressure, coronary perfusion, and ultimately, decreased effectiveness of inotropic therapies on myocardial tissue. Notably, in one study, 70% of patients with isolated hypoperfusion progressed to develop hypotension (classic CS) within 24 h, underscoring the transient nature of compensatory mechanisms [26]. These findings emphasize the need to prioritize early detection of hypoperfusion as a critical feature of ADHF-CS to guide timely intervention and improve outcomes.

## Limitations

This retrospective, observational study has numerous limitations, which include unmeasured confounders, missing data (unrecorded data, inability to determine last dose of prescribed cardiovascular medications, etc.), and selection biases inherent to the study’s methodology. Additionally, the timing of shock recognition was determined retrospectively based on the documentation of SCAI Stage C criteria, which may introduce variability in the exact timing of intervention relative to actual shock onset. Our results should largely be interpreted as hypothesis generating and do not imply causation. The sample size used for this study was small, but the narrow focus on an ADHF-CS was intentional. Because of the smaller sample size, statistically significant findings should be interpreted with caution. Although patients were enrolled from five emergency departments within a single healthcare system, the study’s monocentric design remains a major limitation. Involving multiple independent healthcare centers in future research will be crucial to minimize center-specific biases and enhance generalizability. Furthermore, due to the limited sample size, we were unable to perform a tercile-based analysis of time-to-inotropic therapy. Instead, we pragmatically selected the 8-h cutoff based on observed survival differences. Future larger studies with sufficient power should consider time-stratified analyses to validate these findings. Our discussed findings do have significant biologic plausibility, and a larger, prospective study should be completed to validate whether early inotropic therapies would improve patient mortality and reduce the need for mechanical circulatory support.

## Conclusion

Our findings suggest that inotropic support initiated within 8 h of acute presentation is associated with decreased 28-day mortality for patients with ADHF-related cardiogenic shock. Peripheral perfusion and cardiac output measurement were less frequently quantified within the first 24 h for patients with delayed inotropic support. Using shock classification tools, such as the SCAI or SHARC criteria, may help prospectively identify patients with CS, especially in its early stages. Future research implementing an early, standardized approach to the diagnosis and management of ADHF-CS could further improve patient outcomes.

## Supplementary Information

Additional file 1.

## Data Availability

All original data and materials are kept in a locally managed REDCap database at the University of Pennsylvania. Deidentified data will be made available online once accepted for publication. The limited dataset will be uploaded to the open access Zenodo database found here: 10.5281/zenodo.13804290.

## Abbreviations

ADHF-CS: Acute decompensated heart failure-associated cardiogenic shock
CI: Cardiac index
CRT: Capillary refill time
PAC: Pulmonary artery catheter
SCAI: Society for Cardiovascular Angiography and Interventions
VA-ECMO: Venoarterial extracorporeal membrane oxygenation
VIS: Vasoactive-inotropic score
