# Supplementary material for: Timing of inotropic support is associated with mortality in patients with acute decompensated heart failure-associated cardiogenic shock
Source: Intensive Care Med Exp. 2025 Oct 31;13:111. doi: 10.1186/s40635-025-00806-z (PMC12579036; doi:10.1186/s40635-025-00806-z)

**Supplemental Table 1. Summary of the Society for Cardiovascular Angiography and Interventions (SCAI) Cardiogenic Shock Stages**

(Adapted from Naidu S, et al., J Am Coll Cardiol. 2022; Mar 8;79(9):933-946.

 doi: 10.1016/j.jacc.2022.01.018. Epub 2022 Jan 31.)


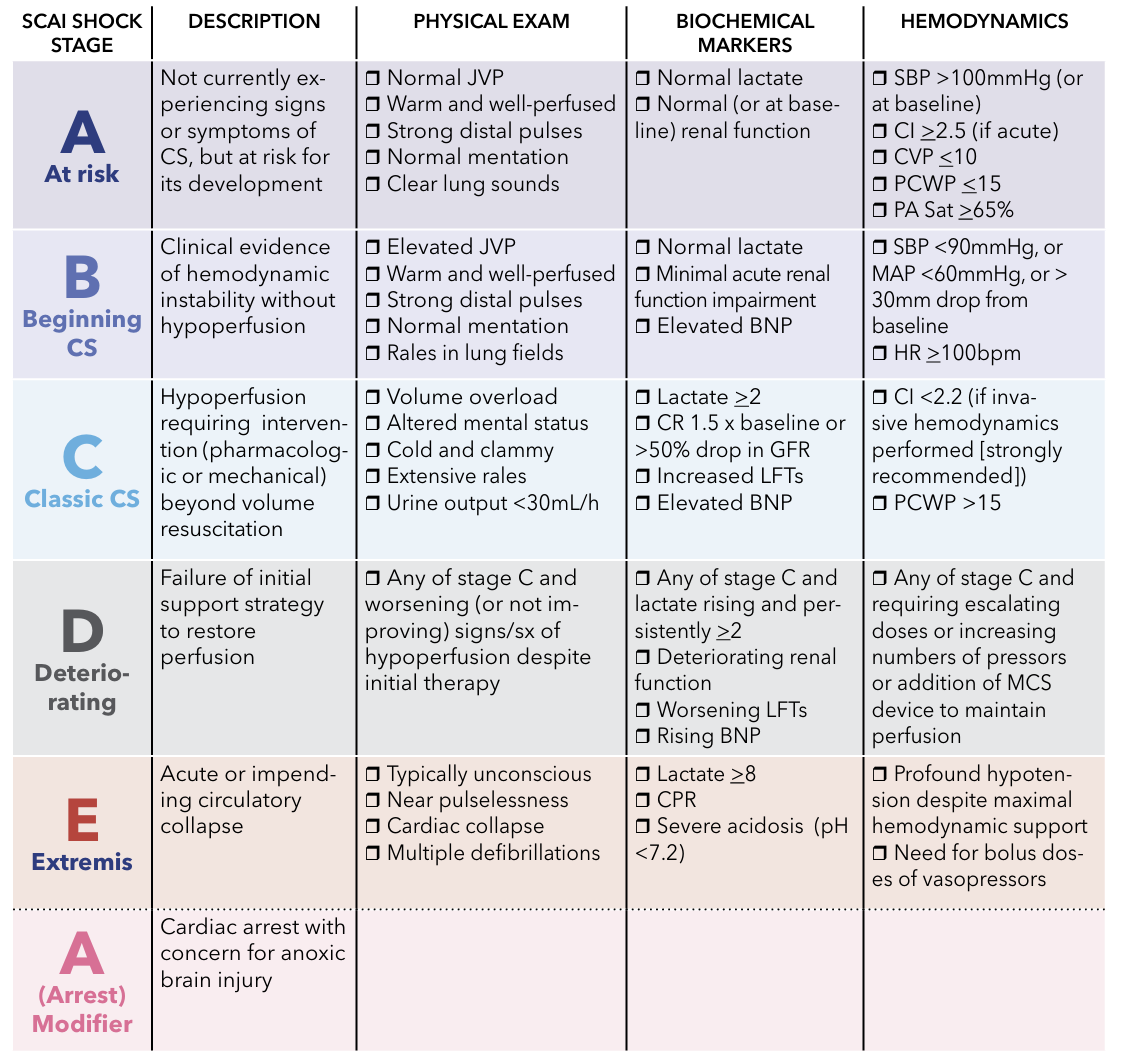

Supplement: Supplementary file 1 — Additional file 1. [file 40635_2025_806_MOESM1_ESM.docx]
